# Supplementary material for: The Effectiveness of Educational Training or Multicomponent Programs to Prevent the Use of Physical Restraints in Nursing Home Settings: A Systematic Review and Meta-Analysis of Experimental Studies
Source: Int J Environ Res Public Health. 2020 Sep 16;17(18):6738. doi: 10.3390/ijerph17186738 (PMC7558973; doi:10.3390/ijerph17186738)
Supplement: Supplementary file 1 [file ijerph-17-06738-s001.pdf]

Figure S1: Prisma check list

Section/topic	#	Checklist item	Reported on page #
**TITLE**			
Title	1	Identify the report as a systematic review, meta-analysis, or both.	1
**ABSTRACT**			
Structured summary	2	Provide a structured summary including, as applicable: background; objectives; data sources; study eligibility criteria, participants, and interventions; study appraisal and synthesis methods; results; limitations; conclusions and implications of key findings; systematic review registration number.	1
**INTRODUCTION**			
Rationale	3	Describe the rationale for the review in the context of what is already known.	1-2
Objectives	4	Provide an explicit statement of questions being addressed with reference to participants, interventions, comparisons, outcomes, and study design (PICOS).	2-3
**METHODS**			
Protocol and registration	5	Indicate if a review protocol exists, if and where it can be accessed (e.g., Web address), and, if available, provide registration information including registration number.	2
Eligibility criteria	6	Specify study characteristics (e.g., PICOS, length of follow-up) and report characteristics (e.g., years considered, language, publication status) used as criteria for eligibility, giving rationale.	2-3
Information sources	7	Describe all information sources (e.g., databases with dates of coverage, contact with study authors to identify additional studies) in the search and date last searched.	2-3
Search	8	Present full electronic search strategy for at least one database, including any limits used, such that it could be repeated.	3
Study selection	9	State the process for selecting studies (i.e., screening, eligibility, included in systematic review, and, if applicable, included in the meta-analysis).	2-3
Data collection process	10	Describe method of data extraction from reports (e.g., piloted forms, independently, in duplicate) and any processes for obtaining and confirming data from investigators.	3-4
Data items	11	List and define all variables for which data were sought (e.g., PICOS, funding sources) and any assumptions and simplifications made.	4-5
Risk of bias in individual studies	12	Describe methods used for assessing risk of bias of individual studies (including specification of whether this was done at the study or outcome level), and how this information is to be used in any data synthesis.	9-10
Summary measures	13	State the principal summary measures (e.g., risk ratio, difference in means).	11-12
Synthesis of results	14	Describe the methods of handling data and combining results of studies, if done, including measures of consistency (e.g., I^2) for each meta-analysis.	16-17

Section/topic	#	Checklist item	Reported on page #
Risk of bias across studies	15	Specify any assessment of risk of bias that may affect the cumulative evidence (e.g., publication bias, selective reporting within studies).	9-10
Additional analyses	16	Describe methods of additional analyses (e.g., sensitivity or subgroup analyses, meta-regression), if done, indicating which were pre-specified.	16-17
**RESULTS**			
Study selection	17	Give numbers of studies screened, assessed for eligibility, and included in the review, with reasons for exclusions at each stage, ideally with a flow diagram.	3-5
Study characteristics	18	For each study, present characteristics for which data were extracted (e.g., study size, PICOS, follow-up period) and provide the citations.	5-8
Risk of bias within studies	19	Present data on risk of bias of each study and, if available, any outcome level assessment (see item 12).	9-10
Results of individual studies	20	For all outcomes considered (benefits or harms), present, for each study: (a) simple summary data for each intervention group (b) effect estimates and confidence intervals, ideally with a forest plot.	9-10
Synthesis of results	21	Present results of each meta-analysis done, including confidence intervals and measures of consistency.	14-17
Risk of bias across studies	22	Present results of any assessment of risk of bias across studies (see Item 15).	9-10
Additional analysis	23	Give results of additional analyses, if done (e.g., sensitivity or subgroup analyses, meta-regression [see Item 16]).	16-17
**DISCUSSION**			
Summary of evidence	24	Summarize the main findings including the strength of evidence for each main outcome; consider their relevance to key groups (e.g., healthcare providers, users, and policy makers).	17-18
Limitations	25	Discuss limitations at study and outcome level (e.g., risk of bias), and at review-level (e.g., incomplete retrieval of identified research, reporting bias).	18
Conclusions	26	Provide a general interpretation of the results in the context of other evidence, and implications for future research.	19
**FUNDING**			
Funding	27	Describe sources of funding for the systematic review and other support (e.g., supply of data); role of funders for the systematic review.	19

From: Moher D, Liberati A, Tetzlaff J, Altman DG, The PRISMA Group (2009). Preferred Reporting Items for Systematic Reviews and Meta-Analyses: The PRISMA Statement. PLoS Med 6(6): e1000097. doi:10.1371/journal.pmed1000097

For more information, visit: **www.prisma-statement.org**.

Table S1: Definition and measured status of restraint in included study

	Conceptual definition of Physical Restraint (PR)	Outcome measures: Physical Restraint
**Abraham et al. (2019)**	"Any action or procedure that prevents a person's free body movement to a position of choice and/or normal access to his/her body by the use of any method, attached or adjacent to a person's body that he/she cannot control or remove easily" (Bleijlevens et al., 2016).<br>Physical restraints such as bed rails, belts, and fixed tables in chairs	Proportion of residents with at least one physical restraint after twelve months. Physical restraint use was assessed through direct observation at three time points: before randomization (T_0), after six months (T_1), and after twelve months (T_2)<br>Observations were performed twice a day (morning and evening)<br>Physical restraints were documented if they were applied at the time of visit. Residents were assessed as having a physical restraint if such a measure was applied at least on one of the two observations points
**Capezuti (2007)**	Side rails are adjustable metal or rigid plastic bars that attach to the bed and come in an assortment of sizes (full-, three-quarter-, half-, and quarter-length rail, split rail configuration, and alternate split rail configuration) and shapes	Observation rounds during the late evening and night shifts (between 9 p.m. and 6 a.m.) on all residents. The observation rounds confirmed the time and the location (e.g., bedroom) and position (e.g., lying in bed) of each resident using the Restraint Use Observation Tool. Side rail use was dichotomized as restrictive or nonrestrictive.<br>Any restraint use (chest/vest, wrist/ankle, belt, or pelvic restraints and geriatric/recliner/wheelchair with fixed tray table) noted on the Minimum Data Set or physician order during the last month of record review was recorded
**Evans et al (1997)**	OBRA '87: restraints included vest/ chest, wris/ankle, mitt, belt, pelvic, or geriatric/reclined wheelchair with fixed tray-table. Siderails were excluded	Two scores were determined: Restraint status (yes, no) indicated whether the resident was ever observed to be restrained over the 72 hours restraint prevalence (number restrained/total number residents **x** 100) were determined from this score for each site and each unit within each site. Restraint intensity indicated the number of times in the 18 observations that the resident was restrained.
**Gulpers et al. (2011; 2012; 2013)**	The use of physical restraints, defined as any limitation in an individual's freedom of movement. Belt use was defined as the restraining of a resident by a belt at least once per day.<br>Physical restraints e.g., wheel, chair with a locked tray table, special sheet, full-enclosure bedrails, chair on a board (chair whose legs are fixed to a board), deep or overturned (wheel)chair, sleep suit.	Use of belt restraints (primary outcome) and other physical restraints (secondary outcomes) was measured per resident using an observation tool developed by Huizing et al. (2006): four times during a 24-h period (morning, afternoon, evening and night. Use of other types of physical restraint simultaneously with belt use was recorded as present or absent, using the same observation tool.
**Huizing et al. (2006,2009a; 2009b)**	Any limitation on an individual's freedom of movement was regarded as a physical restraint.<br>Physical restraints e.g., belts tied to a chair or bed; bilateral bedrails of any length; chairs with fixed tray tables; deep or tipped chairs; chairs on a board (chairs whose legs are fixed to a board) and placed near a table, thus preventing residents from moving the chair and leaving the table; special sheets (special fitted sheets with a coat enclosing the mattress); sleep suits (clothing measure that limit behaviors); sensor mats (including sensor strips in beds); and infrared systems (motion alarms).	An observation tool was designed to record restraint data per resident.<br>Restraint status: indicates whether the resident was observed to be restrained at any time during the four observations over 24 hours (yes/no)<br>Types of physical : absent or present on four separate occasions (morning, afternoon, evening, and night) over 24 hours.<br>Restraint intensity: indicated the number of times in four observations over a 24-h period that a resident was restrained, ranging from not restrained in four observations (score 0) to restrained during all four observations (score 4).<br>The duration of each restraint use (in minutes or hours) was not measured.
**Koczy 2011**	Any device, material or equipment attached to or near a person's body and which cannot be controlled or easily removed by the person and which deliberately prevents or is deliberately intended to prevent a person's free body movement to a position of choice and/or a person's normal access to their body (Joanna Briggs Institute)<br>Only the use of belts tied to a chair or bed and chairs with fixed tables were used for the outcome evaluation. Bed rails were not included.	Daily documentation for each resident (3 days) of PR<br>The duration per day for which restraints were used was documented in a daily calendar
**Kopke (2012)**	Any device, material, or equipment attached to or near the resident's body, which cannot be controlled easily or removed by the person and which deliberately prevents or is deliberately intended to prevent free body movement to a position of choice.	Direct observation at 3 time points during 1 day (morning, noon, evening). All residents with a physical restraint at 1 or more of the 3 time points were counted as having a restraint.
**Pellfolk et al. (2010)**	Any technical device that inhibits a person's free physical movement (e.g., belts and chairs with tables), excluding bedrails	Registered the type of restraint, the reason for its use, and time spent in restraint daily on a form for 3 weeks before and after the intervention. In the analysis, it was dichotomized into restrained at least once during 3 weeks or not at all<br>A control variable concerning restraint use in the preceding week was incorporated into the Multi-Dimensional Dementia Assessment Scale (MDDAS) at follow-up
**Rovner et al (1996)**	definition not reported. PR were secondary outcome	Restraint use was assessed during activity times and on the nursing units. Nursing home staff determined the use of restraints
**Testad et al. (2005)**	Definition not reported	Frequency of use of restraints assessed by a standardized interview (Kirkevold et al., 2004, for details of this interview).
**Testad et al.**	Any limitation on a person's freedom or movement including PR, electronic	Use of restraint was determined by a standardized interview during the last / days was recorded. Item including 14

**(2010)**	surveillance, force or pressure in medical examination or treatment, or any force or pressure in ADL	questions covering PR (5), electronic surveillance (2), use force or pressure medical examination or treatment (2), and use of force or pressure in activities of daily living (ADL, 5)<br>Restraints were coded as "present" or "not present"<br>Restraint use was grouped into structural and interactional
**Testad et al. (2016)**	Restraint may be defined as any limitation on a person's freedom or movement (Hantikainen, 1998). The established international definition of physical restraint (PR) includes any devices, equipment, or aid designed to confine a person's bodily movement (Evansetal.,1997).Restraint may also include confining a person in a locked room, use of electronic surveillance and treatment, or examination against his or her will (Kirkevold et al., 2003).	Use of restraint (at least one episode during 1week) was determined by a standardized interview, including 14 questions covering PR (5), electronic surveillance (2), medical treatment (2), and use of force or pressure in activities of daily living (ADL, 5; Kirkevold et al., 2003).<br>Frequency of use per resident was recorded within a range of at least once a week to several times a day.<br>Restraint use was grouped into structural (seven types, aimed at protecting the resident, such as PR and electronic surveillance) and interactional (seven types, aimed at treatment and care in interaction in care-giving activities).
